# Transactions of the South Carolina Medical Association, at the Extra Meeting in Sumpter, and at the Annual Meeting in Charleston

**Published:** 1857-10

**Authors:** 


*Transactions of the South Carolina Medical Association, at the Extra Meeting in Sumpter, and at the Annual Meeting in Charleston.*

The transactions of the above Association are well arranged in pamphlet form, embracing 64 pages of interesting and important matter. They contain an abstract of the proceedings of the "Board of Counsellors" at several regular and adjourned meetings, and also, the minutes of the extra meeting at Sumpter, and annual meeting at Charleston. The association seems to be in a highly prosperous and flourishing condition, and to have at its foundation the elements of success. The Appendix consists of the principal papers read before the Association, and the By-Laws. Among the papers of most interest, is one entitled, "Observations on the Medical Properties of the Gelseminum Sempervirius—Yellow Jessamine—by J. A. Myers, M. D., from which we extract the following conclusions of the writer.

"I regard it as a direct sedative; more safe and manageable than veratrum viride, and more generally applicable in practice."

"I esteem it a most valuable adjuvant to other treatment in all cases where high arterial action exists, in which it is desirable to lessen the frequency of the pulse, and to calm excitement, and when, as in the case of injuries, it is desirable to lessen the irritability of the nervous system; also, in that troublesome hysterical exaltation of the nervous sensibilities, so often met with in enervated females, its value cannot be too highly estimated."

"In short, it is a specific for no particular disease, but an admirable adjuvant in the treatment of nearly all."

"With its poisonous effects when given in over-doses, I have no acquaintance, but do not doubt the possibility of its doing harm in the hands of the careless."

The oration by Dr. H. Smith, Report of Committee on Disease Tables, Prof. Shephard on Aluminum, and Dr. Ware on Mercury in Typhoid Fever, are all full of interest, and well merit the attention of the profession. We bespeak for them a general perusal.

